# Early prediction of acute gallstone pancreatitis severity: a novel machine learning model based on CT features and open access online prediction platform

**DOI:** 10.1080/07853890.2024.2357354

**Published:** 2024-05-30

**Authors:** Yuhu Ma, Ping Yue, Jinduo Zhang, Jinqiu Yuan, Zhaoqing Liu, Zixian Chen, Hengwei Zhang, Chao Zhang, Yong Zhang, Chunlu Dong, Yanyan Lin, Yatao Liu, Shuyan Li, Wenbo Meng

**Affiliations:** a Department of Anesthesiology, The First Hospital of Lanzhou University, Lanzhou, Gansu, China; b Department of General Surgery, The First Hospital of Lanzhou University, Lanzhou, Gansu, China; c Clinical Research Center, Big Data Center, The Seventh Affiliated Hospital, Sun Yat-sen University, Guangzhou, Guangdong Province, China; d School of Medical Information and Engineering, Xuzhou Medical University, Xuzhou, Jiangsu, China; e Department of Radiology, The First Hospital of Lanzhou University, Lanzhou, Gansu, China; f Department of Orthopedics, The First Hospital of Lanzhou University, Lanzhou, Gansu, China

**Keywords:** Gallstone pancreatitis, CT features, radiomics, random forest, prediction

## Abstract

**Background:**

Early diagnosis of acute gallstone pancreatitis severity (GSP) is challenging in clinical practice. We aimed to investigate the efficacy of CT features and radiomics for the early prediction of acute GSP severity.

**Methods:**

We retrospectively recruited GSP patients who underwent CT imaging within 48 h of admission from tertiary referral centre. Radiomics and CT features were extracted from CT scans. The clinical and CT features were selected by the random forest algorithm to develop the ML GSP model for the identification of severity of GSP (mild or severe), and its predictive efficacy was compared with radiomics model. The predictive performance was assessed by the area under operating characteristic curve. Calibration curve and decision curve analysis were performed to demonstrate the classification performance and clinical efficacy. Furthermore, we built a web-based open access GSP severity calculator. The study was registered with ClinicalTrials.gov (NCT05498961).

**Results:**

A total of 301 patients were enrolled. They were randomly assigned into the training (*n* = 210) and validation (*n* = 91) cohorts at a ratio of 7:3. The random forest algorithm identified the level of calcium ions, WBC count, urea level, combined cholecystitis, gallbladder wall thickening, gallstones, and hydrothorax as the seven predictive factors for severity of GSP. In the validation cohort, the areas under the curve for the radiomics model and ML GSP model were 0.841 (0.757–0.926) and 0.914 (0.851–0.978), respectively. The calibration plot shows that the ML GSP model has good consistency between the prediction probability and the observation probability. Decision curve analysis showed that the ML GSP model had high clinical utility.

**Conclusions:**

We built the ML GSP model based on clinical and CT image features and distributed it as a free web-based calculator. Our results indicated that the ML GSP model is useful for predicting the severity of GSP.

## KEY MESSAGES

1. ML GSP model based on machine learning has good severity discrimination in both training and validation cohorts (0.916 (0.872–0.958), 0.914 (0.851–0.978), respectively).
2. We built an online user-friendly platform for the ML GSP model to help clinicians better identify the severity of GSP.

## Introduction

Gallstones are the main cause of acute pancreatitis (AP), and their incidence is as high as 50% [1]. Cholecystectomy is an important surgical procedure for preventing recurrence and other gallstone-related complications in acute gallstone pancreatitis (GSP) patients [2, 3]. Patients with moderate or severe GSP are generally treated with cholecystectomy after systemic or local complications improve [3, 4]. However, for patients with mild GSP, randomized controlled trials showed that cholecystectomy within 48 h of admission was superior to delayed surgery and reduced the risk of recurrence of biliary complications [5–8]

A critical stage in the management of postadmission GSP is severity evaluation. The bedside index for severity in AP (BISAP) score, modified computed tomography severity index (MCTSI) score, and biochemical indicators are currently widely used in the early prediction of AP severity and are easier and simpler to determine than the Acute Physiology and Chronic Health Examination (APACHE)-II and Ranson’s standard scores [9, 10]. The BISAP score can be immediately evaluated after admission, albeit with low specificity and accuracy [11]. CT plays an important role in evaluating the severity of AP. However, the morphological changes and complications of the pancreas, especially necrosis, in some patients with early stages of disease are not obvious [12, 13]. Per previous studies, up to 15% of people with mild pancreatitis as predicted by the conventional scoring method go on to develop moderate or severe pancreatitis [14, 15]. Therefore, new GSP grading ancillary diagnostic tools should be continuously developed [13].

An innovative analytics method called radiomics uses automated or semiautomated software to extract high-throughput impact features, convert images into high-dimensional data, and use machine learning techniques to combine important imaging histology features and clinical factors to support clinical decision making [16, 17]. Radiomics can capture texture or particle changes as well as pathophysiological features in addition to typical morphological features [18]. Radiomics is widely used to identify tumours, forecast lymph node metastasis, and assess the effectiveness of radio chemotherapy [17, 19, 20]. The latest evidence from nontumor studies, such as those investigating postoperative complications and disease recurrence, has shown that radiomics can produce positive outcomes [21–23]. The gold standard for determining the severity of GSP is still abdominal CT. To our knowledge, the value of CT images, providing quantitative features, such as gallbladder features (stone size and diameter) or pancreatic features (pancreatic duct diameter, pancreatic atrophy), combined with clinical data to assess the severity of pancreatitis is unclear. Therefore, this study aimed to compare the early prediction performance of machine learning models based on CT imaging for GSP severity.

## Methods

Patient studies were conducted according to the guiding principles of the Helsinki Declaration and were registered with Clinicaltrial.gov (NCT05498961). This study was approved by the Ethics Committee of the First Hospital of Lanzhou University (LDYYLL-2022-352). Due to the retrospective nature of the study, written informed consent was abandoned. The study adheres to STROBE guidelines.

### Patients

Patients hospitalized for GSP from January 2016 to January 2022 were identified from the institutional database. GSP diagnostic criteria and severity assessment were based on AP Atlanta criteria revised in 2012 [11]. Supplementary materials S1 and Figure S1 present the inclusion and exclusion criteria as well as the patient selection flowchart. In total, 301 patients were identified, including 173 with mild patients and 129 with severe GSP patients. A random number generated by a computer was allocated to each individual to assign them to the training cohort (*n* = 210) and validation cohort (*n* = 91) at a ratio of 7:3. Laboratory tests parameters were obtained by reviewing patients’ medical records. Supplementary Materials S2–S3 list the evaluation criteria that comprised the severity scoring system for GSP patients. In our study, Mild acute pancreatitis (MAP) was identified as mild, and Moderate to severe acute pancreatitis (MSAP) and Severe acute pancreatitis (SAP) as severe.

### CT image acquisition

No more than 2 days from GSP onset, the CT examination was completed. All examinations were performed with a Sensation 16 CT scanner (Siemens). The scanning energy was 120 kVp, and smart mAs were used. The slice thickness was 4.5 mm, and the pixel spacing was 0.408 × 0.408 cm^2^.

### Evaluation of CT features

Based on the results of a thorough literature search for pancreatic and biliary CT findings in GSP patients and our centre’s experience, the reader evaluated the following indications: (1) gallbladder wall thickening; (2) gallstones (single or multiple); (3) maximal gallbladder diameter; (4) maximal gallstone diameter; (5) maximal bile duct diameter; (6) pancreatic duct diameter (pancreatic head segment); (7) pancreatic atrophy (anteroposterior body diameter less than 20 mm); (8) pancreatic calcifications; (9) duodenal diverticulum; (10) combined acute cholecystitis (evaluation according to Tokyo guidelines); and (11) MCTSI score. All abdominal CT scans were independently reviewed by two experienced doctors - a radiologist with 25 years of clinical experience and a general surgeon with 12 years of clinical experience. Both reviewers were blinded to patient prognosis when reviewing the scans. If there is a disagreement between two readers, it is decided by a senior expert.

### Extraction and stability evaluation of radiomics features

The regions of interest (ROIs) in the CT images were manually delineated using 3D Slicer software(version 5.1) by a surgeon with 12 years of clinical experience, and confirmed by another senior radiologist with 25 years of experience. The pancreatic parenchyma and surrounding encased blood vessels and effusion were delineated, and the blood vessels and bile ducts were excluded. CT images were normalized by centering to the mean standard deviation, resampled to a voxel size of 1.0 mm *1.0 mm *1.0 mm using B-Spline interpolation with grey-level discretized by a fixed bin width of 25 in the histogram. The radiomics features were extracted utilizing the Pyradiomics package (version 3.0.1) in Python(version 3.9.0). Stability evaluation is described in Supplementary Materials S4.

### Radiomics features selection and development of radiomics score

Due to a large number of radiomics features, there may be multiple collinearities. Therefore, features selection is crucial to provide optimal prediction features and avoid overfitting. Radiomics features selection based on the training cohort was divided into two steps. First, the Mann–Whitney U test was used to detect the correlation between each radiomics feature and the severity of GSP in patients, and radiomics features with *p* < 0.05 were selected. Second, least absolute shrinkage and selection operator (LASSO) regression analysis was used to adjust the penalty parameter through 10-fold cross-validation, select the optimal features, and further remove redundant features [24–26]. Each patient’s radiomics score was calculated according to the weighted linear combination of the selected features. Supplementary Materials S5 provide details of selected radiomics features.

### Development and validation of the ML GSP model

The clinical and CT features identified for the training cohort were ranked using the mutual information (MI) method and then added one by one to the RF algorithm to select the feature set with the least numbers and the largest AUC. The hyperparameters of the model were optimized by changing the number of estimators, minimum sample split, minimum sample leaf, and maximum depth and then tested in the validation cohort. Finally, the proposed model, ML GSP, was developed to identify the severity of GSP. The Shapley additive explanation (SHAP) method was used to measure the importance of each variable, quantitatively describing the overall relationship between GSP severity and all variables according to the premodel. To validate the accuracy of the ML GSP model feature selection, we performed univariate and multivariate logistic regression on the blood and CT image features. Furthermore, the ML GSP model was compared with radiomics, BISAP, and MCTSI models. The area under operating characteristic curve (AUC), accuracy, sensitivity, specificity, positive predictive value (PPV), and negative predictive value (NPV) were used to evaluate the predictive efficacy of the models. Calibration curve, decision curve analysis (DCA) and clinical impact curve (CIC) were performed to demonstrate the classification performance and clinical efficacy of the model. Furthermore, we built a web-based open access GSP severity calculator that is available for free at http://cppdd.cn/ML_GSP/. In this study, the modelling process was implemented through the sci-kit-learn library (version 0.19.2) in Python.

### Statistical analysis

R languages (Version 4.0.0) were used for statistical analysis. Continuous variables were reported as the median and interquartile range (IQR) or mean and standard deviation (SD) and were compared using the Mann–Whitney test or Student’s t test. Categorical data, presented as numbers and frequencies (%), were compared using the chi-square test or Fisher’s exact test. Stepwise regression based on the Akaike information criterion minimum was used to select variables. The difference in AUC values between different models was evaluated by the DeLong test. A two-sided P value < 0.05 indicated that the corresponding difference was statistically significant.

## Results

### Patient characteristics

A total of 301 patients with GSP, ranging in age from 12 to 87 years old and including 161 males and 140 females, were included in this study. The patient basic characteristics are shown in Table 1. The optimal critical values for the BISAP and MCTSI scores were 2 and 4 points, respectively. There were no significant differences in basic characteristics between the training and validation cohorts (*p* ≥ 0.05). Between the mild and severe groups, there were statistically significant differences in age, gallbladder wall thickening, gallstones, gallstone diameter, acute cholecystitis, hydrothorax, BISAP, MCTSI score, and radiomics score in the training and validation cohorts (*p* < 0.05). The interobserver and intraobserver reproducibility of radiomics features extraction were satisfactory (intraobserver mean ICC: 0.806, observer mean ICC: 0.772).

**Table 1.** Baseline characteristics in training and validation cohorts.

	Training cohort			Validation cohort			
	Mild (*N* = 119)	Severe (*N* = 91)	*P(Intra)*	Mild (*N* = 53)	Severe (*N* = 38)	*P (Intra)*	*P (Inter)*
*Sex*			0.793			0.987	0.096
Male	53 (44.5%)	38 (41.8%)		28 (52.8%)	21 (55.3%)		
Female	66 (55.5%)	53 (58.2%)		25 (47.2%)	17 (44.7%)		
*Age*			0.236			0.045	0.359
<50 years	58 (48.7%)	36 (39.6%)		32 (60.4%)	14 (36.8%)		
≥50 years	61 (51.3%)	55 (60.4%)		21 (39.6%)	24 (63.2%)		
BMI	23.6 (3.51)	23.2 (3.92)	0.481	23.7 (3.07)	23.8 (4.26)	0.953	0.477
*Gallbladder wall thickening*			<0.001			0.001	0.610
No	40 (33.6%)	7 (7.69%)		17 (32.1%)	1 (2.63%)		
Yes	79 (66.4%)	84 (92.3%)		36 (67.9%)	37 (97.4%)		
*Gallstones*			<0.001			0.060	0.837
Single	66 (55.5%)	19 (20.9%)		27 (50.9%)	11 (28.9%)		
Multiple	53 (44.5%)	72 (79.1%)		26 (49.1%))	27 (71.1%)		
Gallbladder diameter(cm)	70.9 (21.2)	77.0 (23.4)	0.053	73.0 (22.7)	78.1 (20.6)	0.272	0.551
Gallstones diameter(cm)	1.00 [0.60;1.40]	0.70 [0.40;1.30]	0.012	1.10 [0.60;1.40]	0.65 [0.46;1.00]	0.081	0.574
*Combined with AC*			<0.001			<0.001	0.918
No	89 (74.8%)	28 (30.8%)		40 (75.5%)	12 (31.6%)		
Yes	30 (25.2%)	63 (69.2%)		13 (24.5%))	26 (68.4%)		
*Pancreatic atrophy*			0.005			1.000	0.968
No	67 (56.3%)	69 (75.8%)		34 (64.2%)	24 (63.2%)		
Yes	52 (43.7%)	22 (24.2%)		19 (35.8%))	14 (36.8%)		
Pancreatic duct diameter(cm)	2.44 (0.74)	2.53 (0.62)	0.367	2.47 (0.74)	2.56 (0.82)	0.605	0.737
*Pancreatic calcifications*			0.808			0.733	0.373
Yes	82 (68.9%)	65 (71.4%)		39 (73.6%)	30 (78.9%)		
No	37 (31.1%)	26 (28.6%)		14 (26.4%)	8 (21.1%)		
*Duodenal diverticulum*			0.760			0.892	1.000
No	99 (83.2%)	78 (85.7%)		45 (84.9%)	31 (81.6%)		
Yes	20 (16.8%)	13 (14.3%)		8 (15.1%))	7 (18.4%)		
Bile duct diameter (cm)	5.82 (1.80)	6.14 (1.61)	0.177	6.27 (1.16)	6.11 (1.43)	0.832	0.873
*BISAP*			<0.001			<0.001	0.222
<2 score	108 (90.8%)	32 (35.2%)		50 (94.3%)	17 (44.7%)		
≥2 score	11 (9.24%)	59 (64.8%)		3 (5.66%)	21 (55.3%)		
*MCTSI*			<0.001			<0.001	0.663
<4 score	85 (71.4%)	20 (22.0%)		39 (73.6%)	9 (23.7%)		
≥4 score	34 (28.6%)	71 (78.0%)		14 (26.4%)	29 (76.3%)		
*Hydrothorax*			<0.001			0.005	0.866
No	85 (71.4%)	28 (30.8%)		35 (66.0%)	13 (34.2%)		
Yes	34 (28.6%)	63 (69.2%)		18 (34.0%)	25 (65.8%)		
Radiomics score	0.30 (0.16)	0.60 (0.16)	<0.001	0.32 (0.16)	0.57 (0.13)	<0.001	0.266

AC, acute cholecystitis; *P(Intra)* the result of uni-variable analyses between mild and severe groups, *P(inter)* significant difference between training and validation cohort.

### Radiomics features selection and radiomics score development

**Figure 1.**
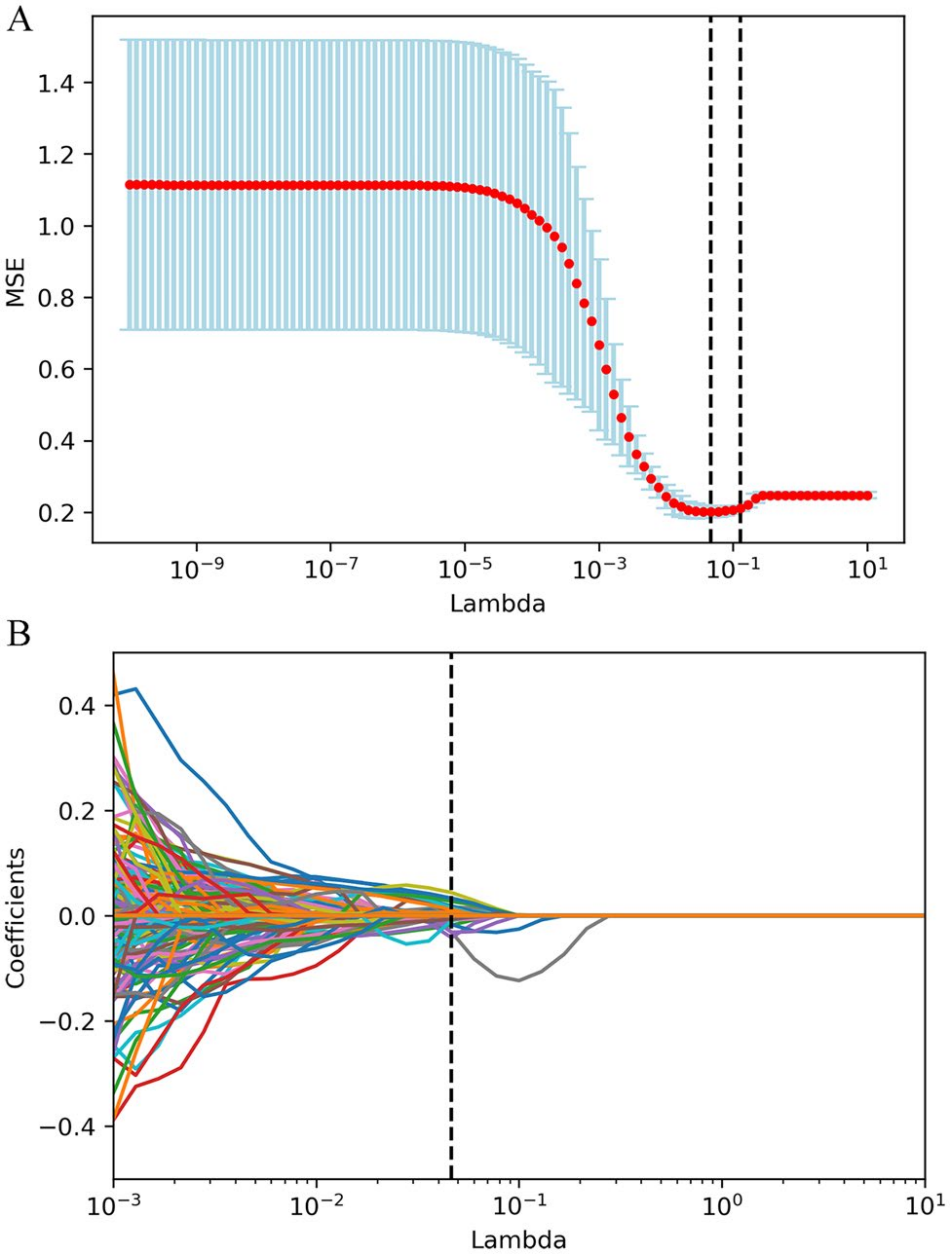
LASSO (least absolute shrinkage and selection operator) regression was used for radiomics features selection. (A), The selection of parameter (λ) in the LASSO model is verified by 10-fold cross-validation via minimum criteria. The relationship between the MSE (misclassification error) curve and λ is plotted. The vertical line is drawn at the optimal value by using the minimum criterion and one standard error of the minimum criterion (1-SE standard). The optimal λ value is 0.464. (B), 862 selected characteristic LASSO coefficient curves. A 10-fold cross-validation is used to draw a vertical line at the selected value, where the best λ produces 29 nonzero coefficients.

In the training cohort, 1171 radiomics features were reduced to 862 features according to the results of the Mann–Whitney U test. LASSO regression resulted in a further reduction to 29 nonzero coefficient characteristics (Figure 1). The radiomics score was calculated using the formula in Supplementary Materials S6. Distributions of radiomics score and GSP severity in the training and validation cohorts are given in Supplemental Figure S2.

### Construction and interpretation of the ML GSP model

**Figure 2.**
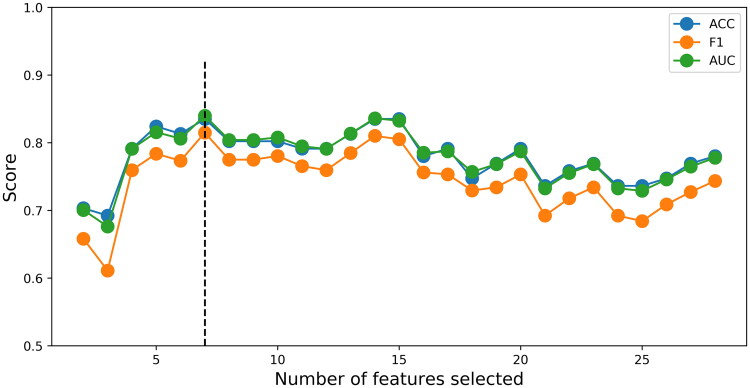
The flowchart of the RF algorithm calculations. With the increase in features, the prediction performance also changes. When the top-ranking 7 features are included (the dotted line), the best AUC of the model is achieved.

**Figure 3.**
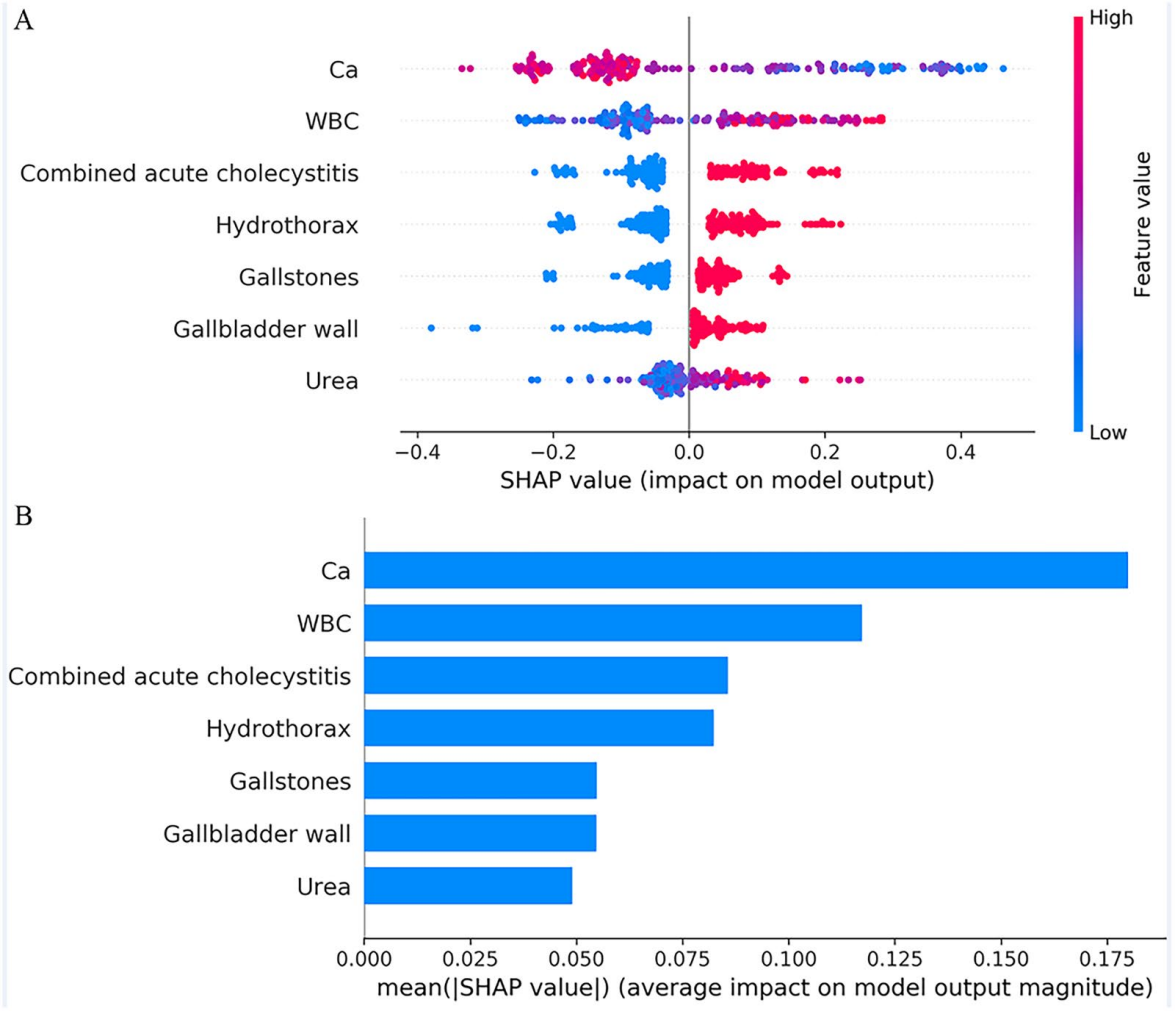
(A) SHAP summary plot of selected variables. Each variable of each patient was coloured by a point according to an attribute value. Red represents a higher value, and blue represents a lower value; (B) shows the importance matrix plot of the ML GSP, describing the importance of each variable in predicting GSP.

All clinical and CT features were analyzed with the RF algorithm, as shown in Figure 2. The critical values for Ca level, WBC count, urea level, combined acute cholecystitis, gallbladder wall thickening, gallstones, and hydrothorax were selected to maximize the AUC, and the ML GSP model was built. SHAP explains the results of the ML GSP model by calculating the contribution of each feature to prediction. The SHAP summary plot and the importance matrix of the ML GSP were shown in Figure 3, which reveals the 7 variables that best predict the severity of GSP according to their importance ranking. Ultimately, the average level of Ca was recognized as the most important predictor. The multivariate logistic regression analysis found that gallbladder wall thickening (OR = 2.224; 95% CI: 1.488–3.325; *p* < 0.001), multiple gallstones (OR = 1.830; 95% CI: 1.317–2.534; *p* < 0.001), combined acute cholecystitis (OR = 2.701; 95% CI: 1.931–3.776; *p* < 0.001), urea level (OR = 1.472; 95% CI: 0.955–2.180; *p* = 0.053), Ca level (OR = 0.479; 95% CI: 0.322–0.713; *p* < 0.001), and hydrothorax (OR = 2.165; 95% CI: 1.564–2.996; *p* < 0.001) were risk factors for the classification of GSP severity (Supplemental Table S1).

### Performance evaluation of models (ML GSP, radiomics, BISAP, and MCTSI model)

**Figure 4.**
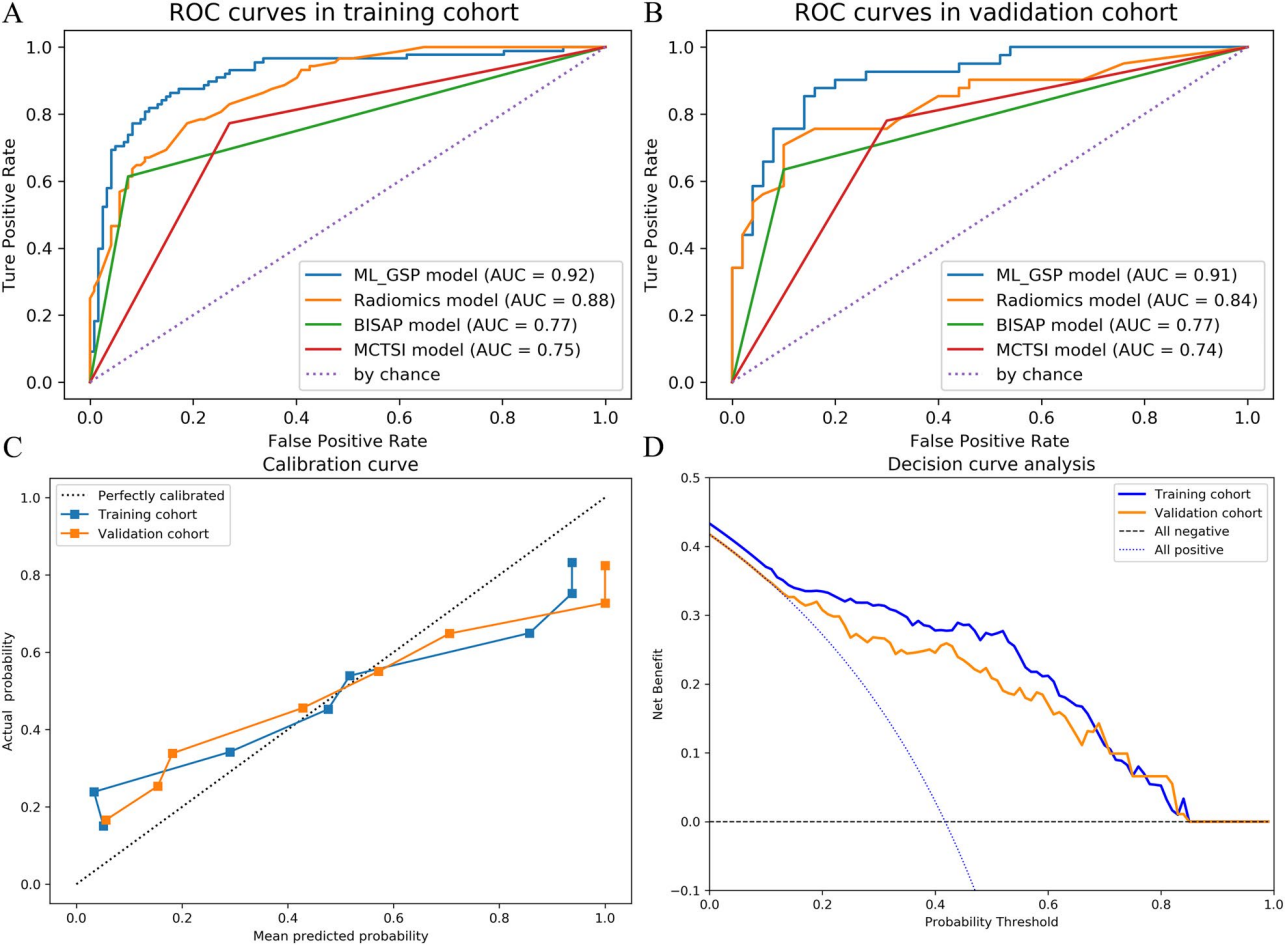
Figure 4. (A) and (B) ROC curves of the ML GSP model, radiomics model, MCTSI model, and BISAP model are shown in the both training and validation cohorts. (C) Calibration curves of the ML GSP model for predicting the severity of GSP between prediction and actual classification in the training cohort and validation cohort. The 45° straight line represents an ideal model perfectly calibrated with an outcome. A closer distance between two curves indicates higher accuracy. (D) Decision curve analysis for the combined ML GSP model in the training cohort and validation cohort. The y-axis shows the net benefit. The x-axis shows the threshold probability. Within reasonable threshold probabilities, combining the RF model in the training and validation cohorts achieves a higher benefit.

A comparison of the clinical models (BISAP and MCTSI), radiomics model, and ML GSP model prediction performance is shown in Table 2. In validation cohorts, the AUCs of the BISAP, MCTSI, radiomics, and ML GSP models were 0.767 (0.667–0.867), 0.744 (0.636–0.844), 0.841 (0.757–0.926), and 0.914 (0.851–0.978), respectively (Figure 4). The CT model (Supplementary Figure S3) that included combined acute cholecystitis, gallbladder wall thickening, and multiple gallstones as factors showed good classification accuracy (AUC: 0.734, 95% CI: 0.624–0.843) in the validation cohort. The DeLong test shows that there is a significant difference in AUC between the radiomics and ML GSP models. The calibration plot shows that the ML GSP model has good consistency between the prediction probability and the observation probability in the training and validation cohorts (Figure 4C). The DCA showed that the ML GSP model provided a better net benefit for GSP with a threshold probability greater than 15% (Figure 4D). A new prediction model was constructed using the risk factors identified by logistic regression. The AUCs for the training and validation cohorts were 0.903 (95% CI 0.860–0.946) and 0.867 (95% CI 0.793–0.941), respectively. Calibration by the Hosmer–Lemeshow test showed that the P values were 0.451 and 0.540 in the training and validation cohorts, respectively. DCA and CIC analysis showed that the model obtained good overall net benefits within the threshold probability range of the training and validation cohorts (Supplementary Figure S4). Although the Logistic regression model had better discriminating power, it was worse than the ML GSP model (validation cohort AUC: 0.914 vs 0.867). This further illustrates the advantages of machine learning in disease classification from the model effect.

**Table 2.** The comparison of four models in the training and validation cohort.

	Model	AUC(95%CI)	Accuracy	Sensitivity	Specificity	NPV	PPV
Training cohort	ML GSP	0.916(0.872–0.958)	0.852	0.909	0.772	0.860	0.847
	Radiomics	0.878(0.826–0.928)	0.800	0.893	0. 633	0.793	0.789
	BISAP	0.770(0.703–0.836)	0.795	0.926	0.613	0.857	0.763
	MCTSI	0.751(0.682–0.819)	0.748	0.729	0.772	0.673	0.816
	CT	0.856(0.803–0.909)	0.795	0.887	0.680	0.831	0.774
Validation cohort	ML GSP	0.914(0.851–0.978)	0.846	0.920	0.756	0.885	0.821
	Radiomics	0.841(0.757–0.926)	0.813	0.830	0.763	0.763	0.830
	BISAP	0.767(0.667–0.867)	0.780	0.900	0.707	0.852	0.789
	MCTSI	0.740(0.636–0.844)	0.736	0.700	0.780	0.680	0.795
	CT	0.734 (0.624–0.843)	0.703	0.875	0.742	0.787	0.844

### Application of the ML GSP model

**Figure 5.**
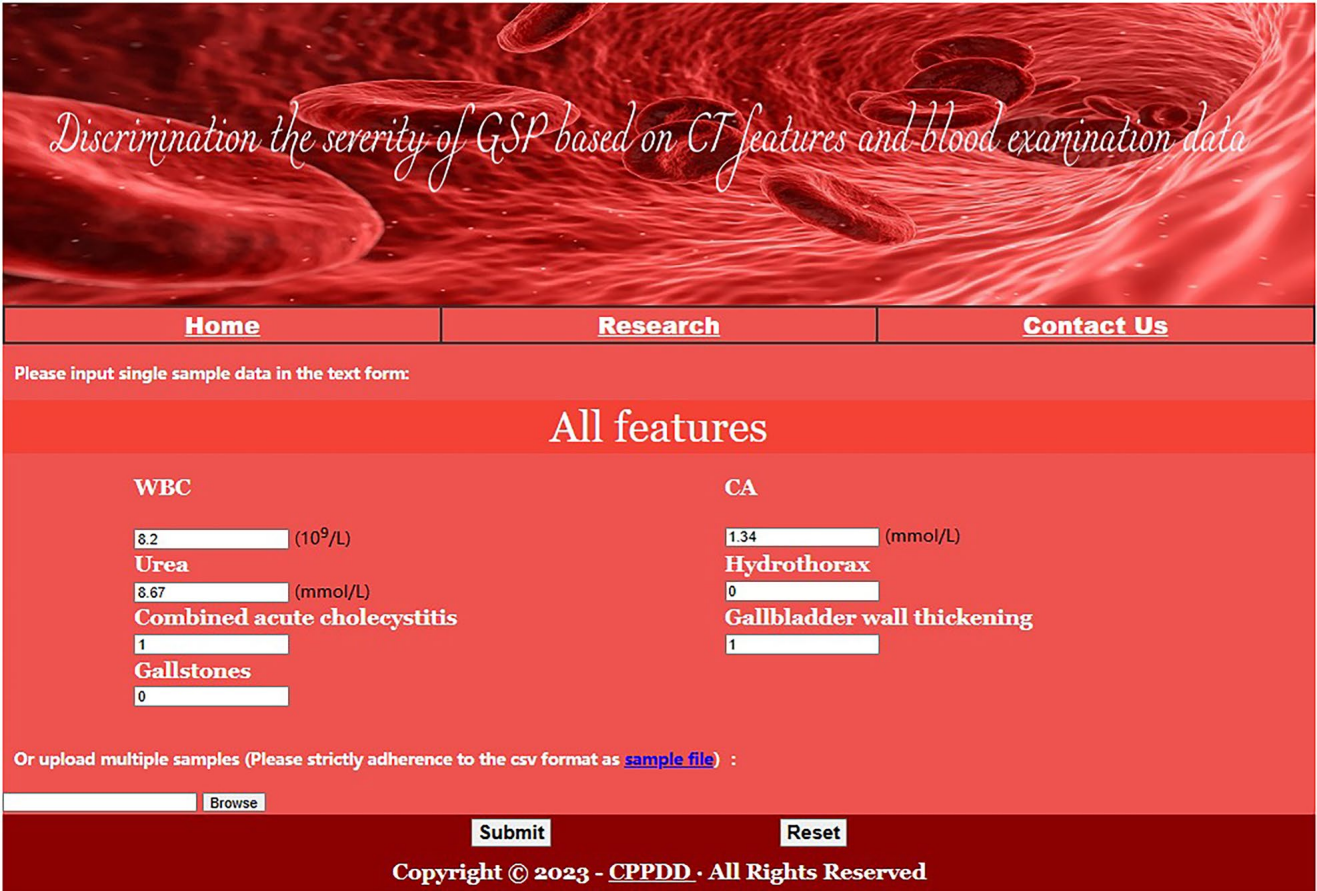
The web prediction tool based on ML GSP model.

To facilitate future application, we created a web server for the ML GSP model in the China Digital Disease Prediction Platform at http://cppdd.cn/ML_GSP/. Users submit values for the 7 features to the corresponding text boxes on the web page, and the calculator shows whether the sample is distinguished with mild or severe GSP (Figure 5).

## Discussion

Assessing the severity of GSP is particularly important for both treatment and avoiding recurrence. In our retrospective study of 301 patients with GSP presenting with a first episode, the ML GSP model (AUC, 0.914) and radiomics model (AUC, 0.841) showed good diagnostic performance in predicting the severity of GSP, better than that of the BISAP (AUC, 0.767) and MCTSI models (AUC, 0.740). Due to the limitations of the clinical application of radiomics, we developed an online user-friendly GSP severity prediction platform for the ML GSP model, which can provide an important reference for subsequent medical decisions regarding GSP patients.

In clinical settings, CT most often determines the severity of GSP by detecting information such as stones in the gallbladder or common bile duct and pancreatic exudate or necrosis [27]. Li et al. showed that duodenal thickening, gallstones, and choledochal ring enhancement were associated with AP prognosis, and the AUC of their proposed CT model was 0.84 [28]. In this study, we evaluated the relationship between GSP severity classification and quantitative features, including properties of the gallbladder, pancreatic atrophy, pancreatic duct diameter, and duodenal diverticulum. Our ML GSP model obtained AUCs of 0.916 (0.872–0.958) and 0.914 (0.851–0.978) in the training and validation cohorts, respectively. The CT feature model showed better discrimination in the validation cohort (AUC: 0.734, 95% CI: 0.624–0.843). Although some features were not statistically significant, this model represents an important method for mining CT information to determine disease classification and prognosis. Notably, the radiomics model had significant sensitivity for both the training (89.3%) and validation cohorts (83.0%). The AUC of the MRI-based radiomics model developed by Lin et al. was 0.848, which also indicates that radiomics has good predictive value in the classification of GSP severity. Some studies have shown that MCTSI and BISAP have similar predictive efficacy for severe pancreatitis [10, 29] and our findings are similar (AUC, 0.767 vs. 0.740).

The quantity and quality of data are essential to the evaluation of radiomics. Our radiomics model showed good classification in predicting the severity of AP, with an accuracy of 81.3% in identifying severe GSP. One factor contributing to the performance reliability was the process of feature selection and model optimization. The Mann–Whitney U test and LASSO regression solved the multicollinearity problem, ensuring the importance of each radiomics features in the model. Another factor may be the heterogeneity of voxel intensity and spatial distribution of texture features [30]. Wang et al. showed that texture features were related to disease heterogeneity (such as micronecrosis and inflammation) [31]. David et al. developed a CT texture model that can identify benign and malignant intraductal papillary mucinous neoplasms (IPMNs) of the pancreas using a logistic regression model on nonenhanced images (AUC, 0.84) [32]. The radiomics score based on 22 texture features in our study also had a high discrimination ability, which may further reflect the macroscopic heterogeneity of texture features in different severities of pancreatitis.

Albasini et al. reported that gallstones are associated with the severity of pancreatitis [33]. Venneman et al. showed that the presence of multiple small stones was positively correlated with the severity of pancreatitis [34]. Our results support previous study findings that multiple gallstones and gallbladder wall thickening were significant risk factors for determining the severity of GSP. We discovered that combined acute cholecystitis was additional supporting evidence for the diagnosis of GSP severity. Hydrothorax and urea levels were key predictors of BISAP score [14]. WBC count as an inflammatory response index of GSP patients has a good contribution to SHAP. We performed multivariate logistic regression analysis to validate the precision of the ML GSP model selection features. The findings revealed that, not only WBC count, but all risk factors showed significant ability to predict GSP severity, further supporting the accuracy of our ML GSP model in discriminating GSP severity based on CT features.

The risk factors and scoring system for evaluating the severity of pancreatitis are complex and difficult to predict even for experienced clinicians. Machine learning methods provide a better means of creating predictive models that can be applied in the clinic. Mutual information can be used in feature selection to measure the mutual dependence between individual features and the target variable. Features can then be ranked based on their mutual information values. In our study, the RF algorithm combined with mutual information method identified WBC count, Ca level, urea level, hydrothorax, combined acute cholecystitis, gallstones, and gallbladder wall thickening as important factors in identifying the severity of GSP, which was validated by multivariate logistic regression. This further demonstrates the accuracy of the ML GSP model in selecting features. The ML GSP model has a sensitivity, specificity, accuracy, and AUC of 0.920, 0.756, 0.846, and 0.914, respectively, showing that the model has a high predictive ability. In addition, we developed a radiomics model to assess the diagnostic value of radiomics. The sensitivity, accuracy, and AUC of the radiomics model were 0.830, 0.813, and 0.841 in the validation cohort, respectively, and its diagnostic efficacy was greater than that of the BISAP and MCTSI scores. For further application, an online user-friendly prediction platform for clinicians worldwide was created for the ML GSP model, and it is freely and openly accessible at http://cppdd.cn/ML_GSP/.

Our study has some limitations. First, any imbalances in the dataset are not compensated for by using any technology (such as oversampling or undersampling). Second, the evaluation of CT features by a radiologist may be a source of bias. Third, our study incorporates wavelet and log features that lack reasonable clinical explanations [35]. Finally, all patients were recruited from a single centre, and patient groups and imaging methods may lead to selection bias. Our findings require an additional prospective study and external validation using sizable multicentre samples, as well as data from many centres and other scanners.

## Conclusions

In our study, we have built an ML GSP model based on CT features and clinical factors and made it available as a free web-based calculator. Our results indicated that the ML GSP model has better performance in predicting the severity of GSP compared to the radiomics model, and can reduce the use of other examinations such as MRI or endoscopic US.

## Supplementary Material

Supplemental Material

## Data Availability

Corresponding authors have accessed and verified the data, and were responsible for the decision to submit the manuscript. All data, study protocol, and statistical analysis plan will be made available upon reasonable request *via* email to corresponding author.
