# Supplementary material for: Early prediction of acute gallstone pancreatitis severity: a novel machine learning model based on CT features and open access online prediction platform
Source: Ann Med. 2024 May 30;56(1):2357354. doi: 10.1080/07853890.2024.2357354 (PMC11141304; doi:10.1080/07853890.2024.2357354)
Supplement: Supplemental Material [file IANN_A_2357354_SM6793.zip › suppl_data/Supplementary material.docx]

**Supplementary material**

**S1. Inclusion and exclusion criteria for all patients**

Inclusion criteria: Patients hospitalized for acute gallstone pancreatitis (GSP) from January 2016 to January 2022.

Exclusion criteria:：(1) Pancreatitis with other causes；(2) No abdominal CT image；(3) History of non-first gallstone pancreatitis；(4) Poor CT imaging quality; (5) More than 48 hours between onset and examination.

**S2. GAP severity evaluation**

Based on the 2012 Atlanta International Consensus, Mild acute pancreatitis (MAP) is defined as no organ failure and no local or systemic complications. Moderate to severe acute pancreatitis (MSAP) refers to patients with organ failure, local complications, or aggravated complications. Severe acute pancreatitis (SAP) is defined as persistent organ failure for more than 48 hours. In our study, MAP was identified as mild and MSAP and SAP as severe.

**S3. Evaluation standards of scoring system (BISAP and MCTSI)**

Without knowing the prognosis, two radiologists with eight and ten years of experience independently scored the severity of GSP through the MCTSI score and reached an agreement through discussion when there was disagreement. BISAP score within 24 hours after admission was calculated by a general surgeon with 12 years of clinical experience.

**S4. Feature stability evaluation**

To reduce the differences in manual segmentation by surgeon, 20 randomly selected computed tomography (CT) images were delineated ROI by a surgeon and senior radiologist. (Reader 1 and Reader 2), and radiomics features were extracted to evaluate the intra-observer and inter-observer reproducibility of the features. To evaluate the consistency within the observer, the reader1 delimited ROI twice a week and extracted radiomics features. Reader 2 delineates ROI and extracts features, and compares the results with the features extracted for the first time by reader 1 to evaluate the reproducibility of features between readers. The intraclass correlation coefficient (ICC) was used to evaluate intra-observer and inter-observer agreement. ICC above 0.75 was considered to have a good consistency. Reader 1 completed the remaining samples.

**S5.** **Radiomics Feature Analysis**

A total of 1171 radiomics features were extracted from each region of interest (ROI). These features include 13 diagnostic features, and 102 original features (composed of six categories of features: first-order statistics, shape-based, gray level co-occurrence matrix

[GLCM], gray level run length matrix [GLRLM], gray level size zone matrix [GLSZM],

and gray level dependence matrix [GLDM]), 352 Laplacian of Gaussian (LOG) features and 704 wavelet features (also composed of six categories of features).

**S6. The formula for radiomics score**

Radiomics score=-0.08440018102409232×diagnostics-Image-interpolatedMean

+0.0008211997213369614×original-shape-MajorAxisLength

+0.0005945509394849939×original-shape-Maximum2DDiameterRow

+(-0.6391256737371769)×log-sigma-2-0-mm-3D-glcm-ClusterShade

+(-0.8006423974836833)×log-sigma-2-0-mm-3D-glcm-MaximumProbability

+(-0.0010761163081803862)×log-sigma-3-0-mm-3D-gldm-DependenceVariance

+(-0.0006519808043732039)×log-sigma-4-0-mm-3D-glcm-ClusterShade

+0.6507550091595316×log-sigma-4-0-mm-3D-glcm-Imc1

+0.3942421925733115×log-sigma-4-0-mm-3D-glszm-SmallAreaLowGrayLevelEmphasis

+(-0.8541623393595789)×log-sigma-5-0-mm-3D-firstorder-InterquartileRange

+(-0.09502297492388798)×log-sigma-5-0-mm-3D-glcm-ClusterShade

+0.0029995566613525725×log-sigma-5-0-mm-3D-glszm-GrayLevelNonUniformity

+0.1276459300913061×log-sigma-5-0-mm-3D-glszm-SmallAreaEmphasis

+11.331238239042705×wavelet-LLH-firstorderMedian

+(-9.900746304785315)×wavelet-LLH-glrlm-GrayLevelNonUniformityNormalized

+3.9412661825865594×wavelet-LHL-firstorder-Median

+0.2801738069253189×wavelet-LHL-glrlm-HighGrayLevelRunEmphasis

+(-3.1854755196060577e-10)×wavelet-LHL-glrlm-LowGrayLevelRunEmphasis

+1.0659659499165968×wavelet-HLL-glcm-Imc2

+(-1.7033609889131511)×wavelet-HLL-glrlm-GrayLevelNonUniformityNormalized

+3.6664212416583993e-07×wavelet-HLL-glrlm-GrayLevelVariance

+0.08854417236397688×wavelet-HLL-glszm-SmallAreaLowGrayLevelEmphasis

+0.6043539798317715×wavelet-HLH-glszm-GrayLevelNonUniformityNormalized

+(-9.200969849884638e-12)×wavelet-HLH-glszm-GrayLevelVariance

+(-6.2595606348189134)×wavelet-HLH-glszm-ZonePercentage

+(-6.227280192680058)×wavelet-HHH-glcm-DifferenceEntropy

+(-1.840380193244112)×wavelet-HHH-glcm-JointEntropy

+(-283.6052124684883)×wavelet-HHH-glrlm-GrayLevelNonUniformityNormalized

+(4.582453974561152e-05)×wavelet-HHH-glrlm-GrayLevelVariance

+156.912697383153

The mind group had low Radiomics score values in both training and validation cohorts.

**Supplementary Figure Legends**

Figure S1 Flowchart of patients enrollment.

Figure S2 Radiomics score for each patient in the training (a) and validation (b) cohorts. GSP severity is marked in different colors.

Figure S3 ROC curves of the CT model were shown in the training and validation cohort, respectively.

Figure S4 ROC curve, calibration curve, clinical decision curve and clinical impact curve based on Logistic regression prediction model. (A) and (B) are ROC curves of the training and validation queues respectively; (C) and (D) are the calibration curves of the training and verification queue. The X-axis represents the predicted probability value, the Y-axis represents the actual probability value, the light blue curve represents the ideal prediction curve, and the black dashed line represents the real prediction performance. (E) and (F) are clinical decision curves for the training and validation cohort, with the x-axis representing predicted probability values and the y-axis representing net benefits. (G) and (H) are clinical impact graphs of the training and validation cohort.

**
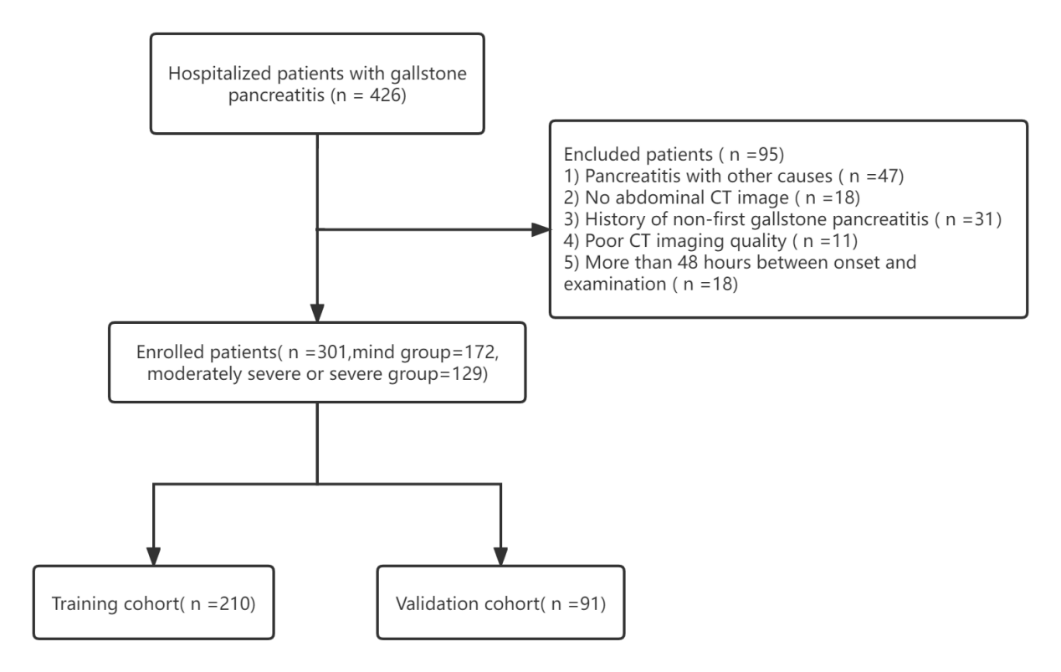
**

Figure S1. Flowchart of patients enrollment.


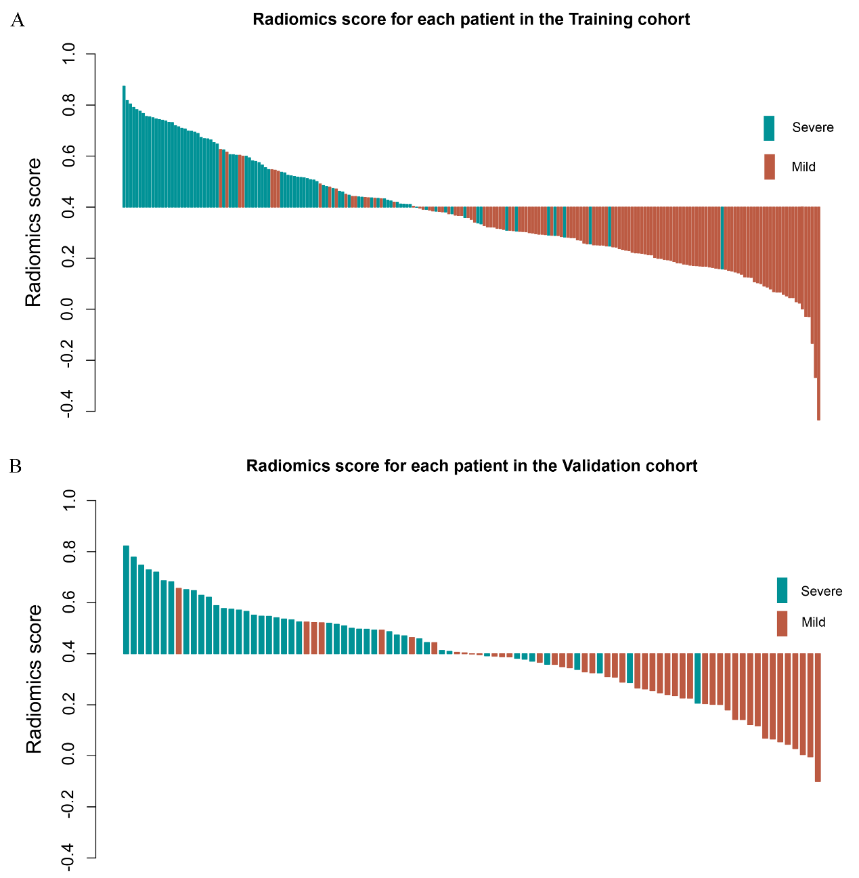


Figure S2. Radiomics score for each patient in the training (A) and validation (B) cohorts. GSP severity is marked in different colors.


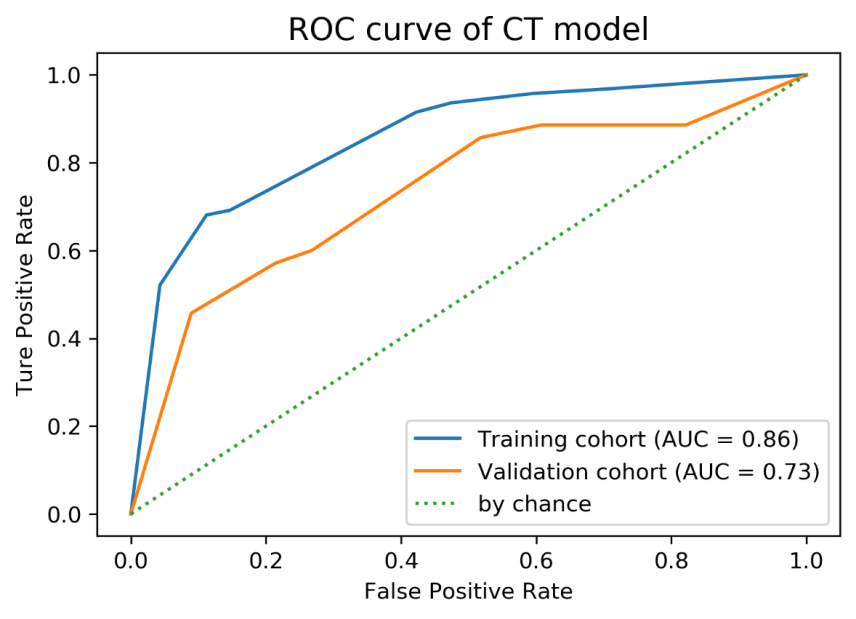


Figure S3 ROC curves of the CT model were shown in the training and validation cohort, respectively.


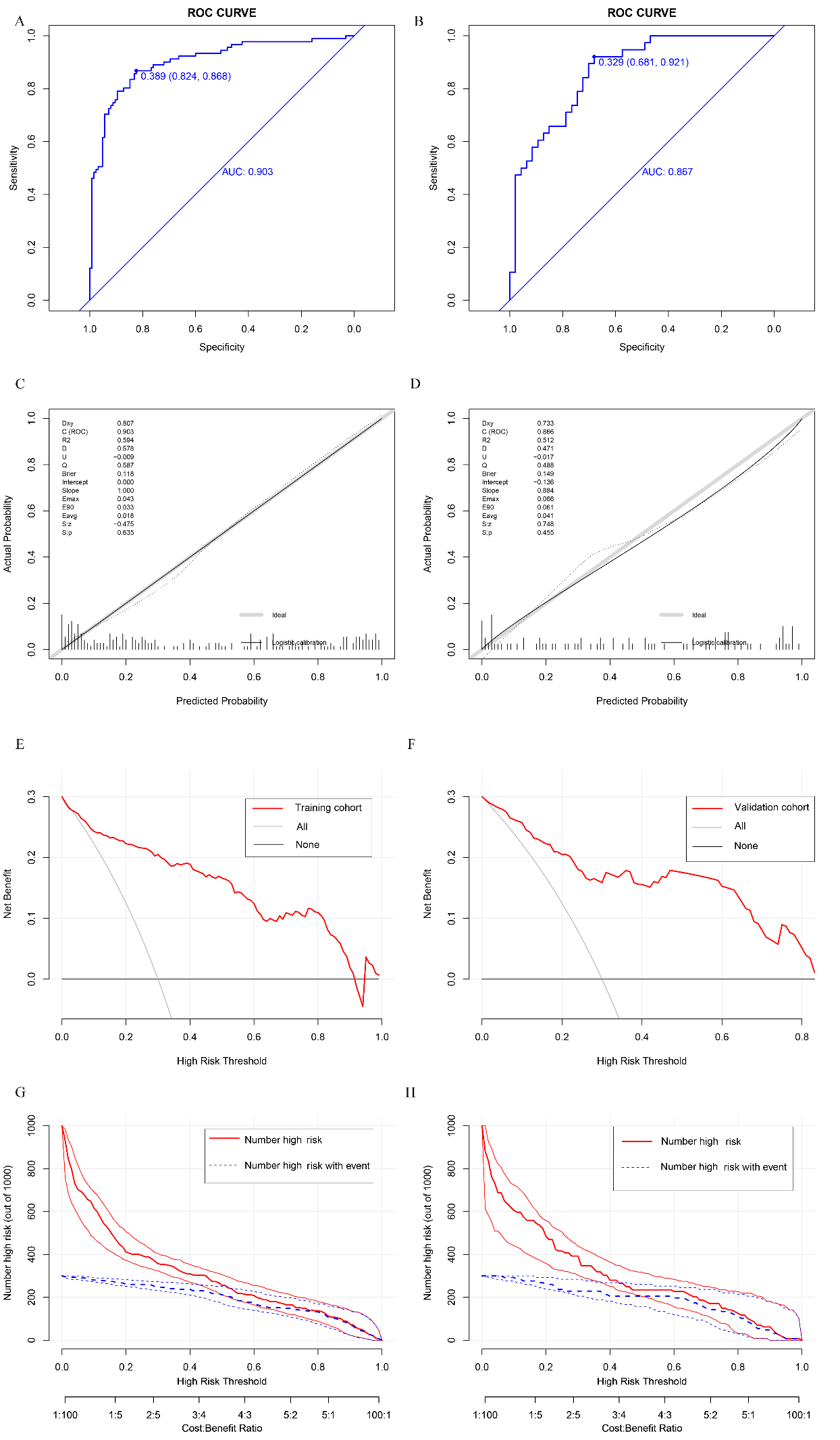


Figure S4 ROC curve, calibration curve, clinical decision curve and clinical impact curve based on Logistic regression prediction model. (A) and (B) are ROC curves of the training and validation queues respectively; (C) and (D) are the calibration curves of the training and verification queue. The X-axis represents the predicted probability value, the Y-axis represents the actual probability value, the light blue curve represents the ideal prediction curve, and the black dashed line represents the real prediction performance. (E) and (F) are clinical decision curves for the training and validation cohort, with the x-axis representing predicted probability values and the y-axis representing net benefits. (G) and (H) are clinical impact graphs of the training and validation cohort.
