# Supplementary material for: Early prediction of acute gallstone pancreatitis severity: a novel machine learning model based on CT features and open access online prediction platform
Source: Ann Med. 2024 May 30;56(1):2357354. doi: 10.1080/07853890.2024.2357354 (PMC11141304; doi:10.1080/07853890.2024.2357354)
Supplement: Supplemental Material [file IANN_A_2357354_SM6793.zip › suppl_data/Supplementary Table 1.docx]

Supplementary Table 1. Univariate and multivariate logistic regression analysis for GSP severity.

|  | **Univariate analyses** | | | **Multivariate analyses** | | |
| --- | --- | --- | --- | --- | --- | --- |
| **Variables** | ***OR*** | **95％ *CI*** | ***P*** | ***OR*** | **95％ *CI*** | ***P*** |
| **Sex (Female)** | 0.973 | 0.774-1.223 | 0.815 |  |  |  |
| **Age (≥50 years)** | 1.316 | 1.044-1.658 | 0.020 |  |  |  |
| **BMI** | 0.982 | 0.922-1.045 | 0.560 |  |  |  |
| **Gallbladder wall (＞3mm)** | 2.291 | 1.660-3.161 | ＜0.001 | 2.224 | 1.488-3.325 | ＜0.001 |
| **Gallstones (Multiple)** | 1.949 | 1.519-2.500 | ＜0.001 | 1.830 | 1.317-2.543 | ＜0.001 |
| **Gallbladder diameter** | 1.316 | 1.044-1.658 | 0.026 |  |  |  |
| **Gallstones diameter** | 0.800 | 0629-1.017 | 0.068 |  |  |  |
| **Combined AC (Yes)** | 2.565 | 1.993-3.301 | ＜0.001 | 2.701 | 1.931-3.776 | ＜0.001 |
| **Pancreatic atrophy (Yes)** | 0.751 | 0.594-0.950 | 0.017 |  |  |  |
| **Pancreatic duct diameter (cm)** | 1.126 | 0.896-1.415 | 0.307 |  |  |  |
| **Pancreatic calcifications（Yes）** | 0.929 | 0.738-1.168 | 0.530 |  |  |  |
| **Duodenal diverticulum (Yes)** | 0.978 | 0.778-1.231 | 0.856 |  |  |  |
| **Bile duct diameter (cm)** | 1.067 | 0.846-1.346 | 0.580 |  |  |  |
| **WBC** | 2.962 | 2.187-4.012 | ＜0.001 |  |  |  |
| **NEUT (％)** | 3.170 | 2.228-4.508 | ＜0.001 |  |  |  |
| **TBIL** | 1.129 | 0.898-1.418 | 0.296 |  |  |  |
| **DBIL** | 1.307 | 1.015-1.683 | 0.038 |  |  |  |
| **HGB** | 0.947 | 0.754-1.190 | 0.643 |  |  |  |
| **HCT** | 0.841 | 0.668-1.059 | 0.141 |  |  |  |
| **ALP** | 1.016 | 0.809-1.276 | 0.890 |  |  |  |
| **GGT** | 1.168 | 0.930-1.468 | 0.182 |  |  |  |
| **Crea** | 1.262 | 0.997-1.598 | 0.053 |  |  |  |
| **Urea** | 2.104 | 1.534-2.885 | ＜0.001 | 1.472 | 0.995-2.180 | 0.053 |
| **Ca^+^** | 0.326 | 0.265-0.495 | ＜0.001 | 0.479 | 0.322-0.713 | ＜0.001 |
| **TC** | 0.681 | 0.536-0.866 | 0.002 |  |  |  |
| **TG** | 0.955 | 0.759-1.202 | 0.701 |  |  |  |
| **HDL-C** | 0.644 | 0.501-0.827 | 0.001 |  |  |  |
| **LDL-C** | 0.730 | 0.577-0.925 | 0.009 |  |  |  |
| **Hydrothorax (Yes)** | 2.221 | 1.737-2.841 | ＜0.001 | 2.165 | 1.564-2.996 | 0.001 |

WBC, White blood count, NEUT**％,** neutrophil percentage, HGB, hemoglobin, HCT, hematocrit, HCT, ALP, alkaline phosphatase, GGT, γ-glutamyl transpeptadaseTBIL, total bilirubin, DBIL Crea, creatinine, TC, total cholesterol, TG, triglyceride, HDL-C, high-density lipoprotein cholesterol, LDL-C, low-density lipoprotein cholesterol.
